# The Intestinal Diseases of Infancy and Childhood

**Published:** 1889-03

**Authors:** 


*The Intestinal Diseases of Infancy and Childhood.* By A. JACOBI, M.D. Detroit: GEORGE S. DAVIS. 1887.

This is one of the best—perhaps, taken altogether, the best—of the many books written on the feeding of young children, and the troubles connected therewith.

It differs from most of its congeners, in being marked by much originality and daring. The author has no regard for ancient fictions or, as some people call them, beliefs; he brings everything to the test of the touchstone of modern experience, and that mostly his own. Dr. Jacobi is a keen observer, and about the most trustworthy of the counsellors who proffer advice in this often difficult question of artificial feeding.

Mothers and nurses have been long encouraged by doctors to feel themselves secure in the idea—delusive more often than not—that their children were being fed with milk from a single cow. A milk-boy, asked by one of his fellows why he was filling a small can from his large one, replied, "The lady at No. 10 always has the milk from one cow." Dr. Jacobi says, "The baby fed on the milk of one cow is, as it were, an appendage of that cow, and dependent upon the animal, and the practice of relying on the milk of one cow is inferior to making use of the mixed milk of a whole dairy."

The author speaks somewhat disparagingly of condensed milk, allowing it only when the choice is between that and bad milk. Instances of the benefit and comfort of using condensed milk must, however, be familiar to every practitioner.

Whilst there are many children who seem to thrive upon almost anything, there are a large number with whom nothing appears to agree for long together. Here again Dr. Jacobi is most helpful. But he is scarcely emphatic enough about the virtues of peptonised milk, merely saying: "Fairchild's method of peptonizing milk is generally understood all over the country, and is widely appreciated." Contrary to the usual belief, he states that "some starch is digested at the very earliest age." His observations about the employment of patented foods are charmingly outspoken and intelligent.

Rather more than the latter half of the book is taken up with the account and treatment of definite intestinal disorders. Here the reader will find much valuable advice.

We recommend Dr. Jacobi's book very heartily.

